# Human Muscle Protein Synthetic Responses during Weight-Bearing and Non-Weight-Bearing Exercise: A Comparative Study of Exercise Modes and Recovery Nutrition

**DOI:** 10.1371/journal.pone.0140863

**Published:** 2015-10-16

**Authors:** Stefan M. Pasiakos, Holly L. McClung, Lee M. Margolis, Nancy E. Murphy, Gregory G. Lin, Jay R. Hydren, Andrew J. Young

**Affiliations:** 1 Military Nutrition Division, US Army Research Institute of Environmental Medicine, Natick, MA, United States of America; 2 Military Performance Division, US Army Research Institute of Environmental Medicine, Natick, MA, United States of America; University of Birmingham, UNITED KINGDOM

## Abstract

Effects of conventional endurance (CE) exercise and essential amino acid (EAA) supplementation on protein turnover are well described. Protein turnover responses to weighted endurance exercise (i.e., load carriage, LC) and EAA may differ from CE, because the mechanical forces and contractile properties of LC and CE likely differ. This study examined muscle protein synthesis (MPS) and whole-body protein turnover in response to LC and CE, with and without EAA supplementation, using stable isotope amino acid tracer infusions. Forty adults (mean ± SD, 22 ± 4 y, 80 ± 10 kg, VO_2peak_ 4.0 ± 0.5 L∙min^-1^) were randomly assigned to perform 90 min, absolute intensity-matched (2.2 ± 0.1 VO_2_ L∙m^-1^) LC (performed on a treadmill wearing a vest equal to 30% of individual body mass, mean ± SD load carried 24 ± 3 kg) or CE (cycle ergometry performed at the same absolute VO_2_ as LC) exercise, during which EAA (10 g EAA, 3.6 g leucine) or control (CON, non-nutritive) drinks were consumed. Mixed-muscle and myofibrillar MPS were higher during exercise for LC than CE (mode main effect, *P* < 0.05), independent of dietary treatment. EAA enhanced mixed-muscle and sarcoplasmic MPS during exercise, regardless of mode (drink main effect, *P* < 0.05). Mixed-muscle and sarcoplasmic MPS were higher in recovery for LC than CE (mode main effect, *P* < 0.05). No other differences or interactions (mode x drink) were observed. However, EAA attenuated whole-body protein breakdown, increased amino acid oxidation, and enhanced net protein balance in recovery compared to CON, regardless of exercise mode (*P* < 0.05). These data show that, although whole-body protein turnover responses to absolute VO_2_-matched LC and CE are the same, LC elicited a greater muscle protein synthetic response than CE.

## Introduction

Conventional endurance (CE) exercise models (e.g., cycle ergometry and treadmill) are commonly used to assess the combined effects of endurance-type exercise and essential amino acid (EAA) supplementation on whole-body and skeletal muscle protein turnover. Studies have shown that EAA supplementation spares whole-body protein and enhances skeletal muscle protein synthesis (MPS) in recovery from CE [1,2,3]. However, other studies have failed to observe this effect [4], and whether the effects of CE and EAA supplementation reflect whole-body protein turnover and MPS responses to real-world sporting events, occupational tasks, or exercise training scenarios that include sustained and/or repeated bouts of weighted endurance-type exercise (i.e., load carriage, LC) have not been determined [5]. LC is a unique form of exercise, in that the bioenergetics demands of conventional endurance exercise (CE) are coupled with generation of contractile forces characteristic of resistive-type exercise. Metabolic and mechanical strains produced by LC can exceed CE [6]. Military personnel are an example of individuals commonly tasked to perform LC during training and combat operations, and the loads carried (comprised of body armor, weighted packs, food, water, weapons, etc.) can be substantial, ranging from ~30–60 kg [7]. Thus, carrying heavy loads for prolonged periods may be detrimental to whole-body and skeletal muscle protein retention [5,8,9].

Whole-body protein breakdown and amino acid oxidation may be higher in response to LC than CE, to support higher energy demand and amino acid requirements for whole-body protein synthesis during and after LC [10]. The mechanical strain produced with LC may also elicit MPS responses that differ from CE. CE primarily stimulates the synthesis of non-myofibrillar proteins associated with aerobic adaptations to exercise [11]. We suspect that LC would elicit MPS patterns that resemble those produced in response to either resistance exercise [12] or concurrent resistance and CE training [13,14] and, thus, exceed the MPS response to CE. However, whether LC elicits MPS and whole-body protein turnover responses that differ from CE, and whether those responses augment the synthetic and protein-sparing stimulatory effects of consuming an optimal dose of EAA has not been determined.

The objectives of this randomized, double-blind, placebo controlled trial were to compare MPS and whole-body protein turnover responses to sustained LC and absolute intensity-matched CE. Protein turnover responses to EAA supplementation were also studied to determine whether the effects of consuming EAA are the same during LC as during CE [1]. Our intent was to examine MPS and whole-body protein turnover in response to a military-like, occupational task and to determine whether coupling LC with a previously published, eat-on-the-move, pulsed EAA (and/or protein) feeding strategy provides the same protein synthetic advantages demonstrated in our previous study using a CE model [1]. We hypothesized that LC would elicit a more pronounced myofibrillar MPS response compared to CE, but that sarcoplasmic MPS and whole-body protein turnover would be similar between LC and CE when the absolute exercise intensities are matched. We expected that consuming EAA during LC and CE would enhance the synthetic response, and we hypothesized that the mechanical strain generated with LC would potentiate the protein synthetic response to EAA.

## Materials and Methods

### Volunteer characteristics and experimental design

Forty free-living adults (37 males and 3 females) participated in this study after providing informed, written consent. Volunteers were required to be between the ages of 18–39 years, weight stable (± 2 kg for a period of 2 months), physically fit (peak oxygen uptake, VO_2peak_ 40–60 mL∙kg^-1^∙min^-1^), and have a body mass index (BMI) between 22–29 kg/m^2^. Prospective volunteers reporting metabolic or cardiovascular abnormalities, musculoskeletal injuries, specific food allergies, or the use of medications or nutritional supplements known to influence protein metabolism were excluded from participation. Investigators adhered to the policies for protection of human subjects as prescribed in Army Regulation 70–25 and research was conducted in adherence with the provisions of 32 CFR Part 219, approved by the Institutional Review Board at the US Army Research Institute of Environmental Medicine, Natick, MA.

Height was measured in duplicate to the nearest 0.1 cm using an anthropometer (Item No. 101, Seritex, Inc., Carlstadt, NJ). Body mass was measured to the nearest 0.1 kg after an overnight fast using a calibrated digital scale (WB-110A, Tanita, Tokyo, Japan). Body composition was assessed at baseline using dual energy X-ray absorptiometry (Lunar IDXA, GE Lunar Corporation, Madison, WI). A progressive intensity treadmill test was used to determine VO_2peak_ using indirect calorimetry (TrueOne^®^ 2400 Metabolic Measurement System, ParvoMedics, Sandy, UT). Volunteers were then randomly assigned to one of four experimental groups, each of whom performed a single 90 min exercise bout. Two groups performed CE and the other two performed LC. One of each of the exercise groups received EAA drinks to consume during exercise, and the other groups received control (CON) drinks. MPS was assessed during exercise and recovery and whole protein turnover was determined in recovery only. A resting MPS measure was not included in this study, given MPS responses to endurance-type exercise (i.e., as they relate to resting MPS) are well established [15]. It is also important to note that our intent was not to determine temporal changes in MPS within an exercise mode (with or without EAA), but to examine MPS responses between LC and CE during exercise and recovery independently.

### Diet and physical activity

Volunteers completed 3 d diet and activity records at baseline, and similar to our previous work [1], these records were used to individually prescribe 7 d lead-in diets to maintain body weight and to limit the potential confounding effect of diet on outcome measures. Compliance was confirmed by 24 h dietary recalls conducted every two days during the lead-in phase (Food Processor SQL^®^, version 10, ESHA Research, Salem, OR) (Table 1). Volunteers were also instructed to maintain activity levels reported at baseline for the first five days of the lead-in phase. All resistive and endurance-type activity was prohibited 48 h before data collection to minimize any potential residual effects of previous exercise on protein turnover.

**Table 1:** Baseline and 7 day lead-in dietary intake.

	Energy (kcal∙d^-1^)	Carbohydrate	Fat (g∙kg^-1^∙d^-1^)	Protein
Baseline				
CE				
CON	2800 ± 784	4.6 ± 1.4	1.3 ± 0.4	1.7 ± 0.7
EAA	2845 ± 733	4.1 ± 1.5	1.2 ± 0.4	1.8 ± 0.5
LC				
CON	3211 ± 1057	5.0 ± 1.7	1.5 ± 0.5	1.8 ± 0.6
EAA	3670 ± 709	5.6 ± 1.7	1.6 ± 0.5	1.8 ± 0.5
7 day lead-in				
CE				
CON	2634 ± 119	4.7 ± 1.1	1.2 ± 0.4	1.6 ± 0.3
EAA	2637 ± 153	4.1 ± 1.1	1.1 ± 0.4	1.4 ± 0.2
LC				
CON	2374 ± 473	4.0 ± 1.0	1.1 ± 0.3	1.4 ± 0.4
EAA	2785 ± 503	4.7 ± 1.0	1.2 ± 0.2	1.4 ± 0.3

Data are mean ± SD, n = 10 per group. Dietary intake was assessed using 24 h recalls (Food Processor SQL^®^ (version 10.0, ESHA Research, Salem, OR). CE, conventional endurance exercise; LC, load carriage; CON, control; and EAA, essential amino acids.

### Load carriage and conventional endurance exercise

LC was performed by walking on a treadmill while wearing a weighted vest equivalent to 30% of baseline body mass. CE was non-weight bearing and performed on a cycle ergometer (Lode, BV, Netherlands) to allow for comparisons with our previous studies [1,16]. Baseline VO_2peak_ and associated heart rates at maximal and submaximal levels were used to establish target exercise intensities for the LC and CE trials. Speed and grade for LC and power (watts) for CE were adjusted to match the absolute exercise intensity (intended oxygen uptake was 2.4 L∙m^-1^) and to elicit a similar energy cost (intended energy expenditure was 1050 kcal∙90 min^-1^) between LC and CE. Matching the intensity and energy cost was done to isolate the effects of the possible differences in mechanical force and contractile properties of LC and CE from the relative intensity and energy cost of the exercise bout. A familiarization trial was conducted to ensure the accuracy of the exercise prescription and the ability of the volunteer to complete the prescribed exercise bout. Heart rate was monitored continuously and indirect calorimetry (ParvoMedics, Sandy, UT) was used to verify exercise intensity (15 min intervals) during the familiarization trial and workloads were adjusted to maintain the desired exercise intensity.

### Muscle protein synthesis and whole-body protein turnover assessment

**Fig 1:**
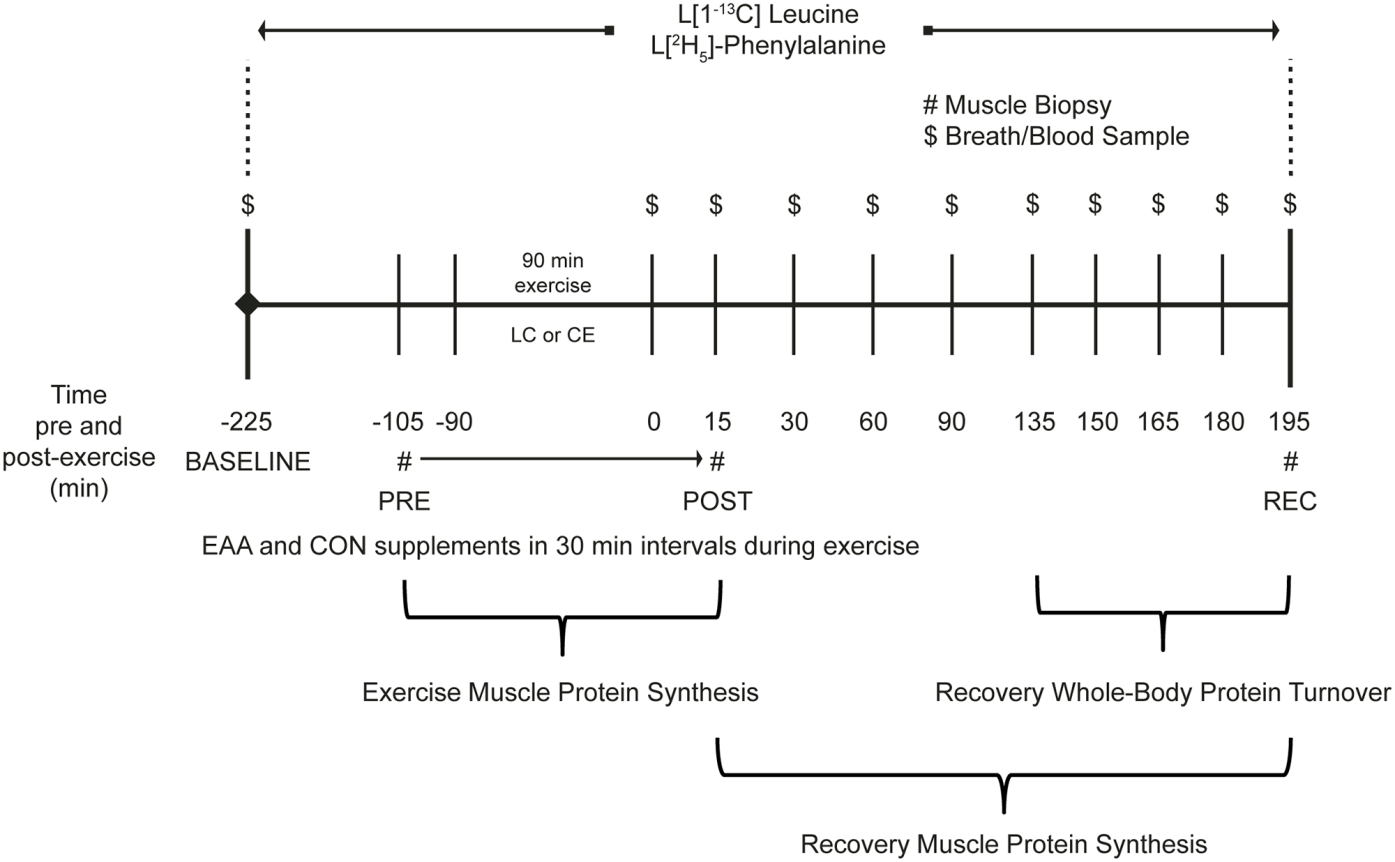
Experimental design. Load carriage (LC) and conventional endurance (CE) exercise muscle protein synthesis and whole-body protein turnover protocols.

Exercise and recovery MPS responses to LC, with and without EAA supplementation, were determined using primed, constant infusions of L-[^2^H_5_]-phenylalanine (2.8 mol∙kg^-1^; 0.07 mol∙kg^-1^∙min^-1^). Whole-body protein turnover was assessed in recovery using primed, constant infusions of L-[1-^13^C]-leucine (7.6 μmol∙kg^-1^; 7.6 μmol·kg^-1^∙h^-1^). Volunteers received a bolus injection of ^13^C-bicarbonate (2.35 μmol∙kg^-1^) before starting the primed, constant infusion of L-[1-^13^C]-leucine to prime the bicarbonate pool. Baseline blood and breath samples were collected to correct for background isotopic enrichments before stable isotope infusions were initiated (Fig 1). Protein turnover studies were conducted between 0600 and 1300 h, after a 12 h fast. Isotopes were commercially available (Cambridge Isotope Laboratories, Andover, MA) and certified sterile and pyrogen free before administering.

After a 2 h isotope equilibration and incorporation period, and before starting the LC or CE exercise bout, a baseline (PRE) percutaneous muscle biopsy was taken from the vastus lateralis [16,17]. Volunteers then began the 90 min, metabolically-matched LC or CE exercise bout. Exercise intensity was verified (and adjusted accordingly) every 30 min adjustments were made based on indirect calorimetry (ParvoMedics, Sandy, UT). There were no differences in absolute (or relative) exercise intensity between groups (Table 2).

**Table 2:** Exercise intensity and total weight carried during the 90 min conventional endurance exercise and load carriage exercise bouts.

	CE		LC	
	CON	EAA	CON	EAA
VO_2_ (L∙min^-1^)	2.2 ± 0.2	2.2 ± 0.1	2.2 ± 0.2	2.2 ± 0.1
Energy (kcal∙90 min^-1^)	977 ± 66	1008 ± 54	1008 ± 71	1002 ± 53
Percent VO_2peak_ (%)	57 ± 5	55 ± 7	58 ± 5	54 ± 6
Load carried (kg)	n/a	n/a	23 ± 3	24 ± 3
Treadmill speed (miles∙h^-1^)	n/a	n/a	3.5 ± 0.2	3.5 ± 0.2
Treadmill incline (%)	n/a	n/a	5.5 ± 1.4	5.1 ± 1.5
Power (watts)	140 ± 8	139 ± 5	n/a	n/a

Data are mean ± SD, n = 10 per group. ^2^CE, conventional endurance exercise; LC, load carriage; CON, control; and EAA, essential amino acids.

**Fig 2:**
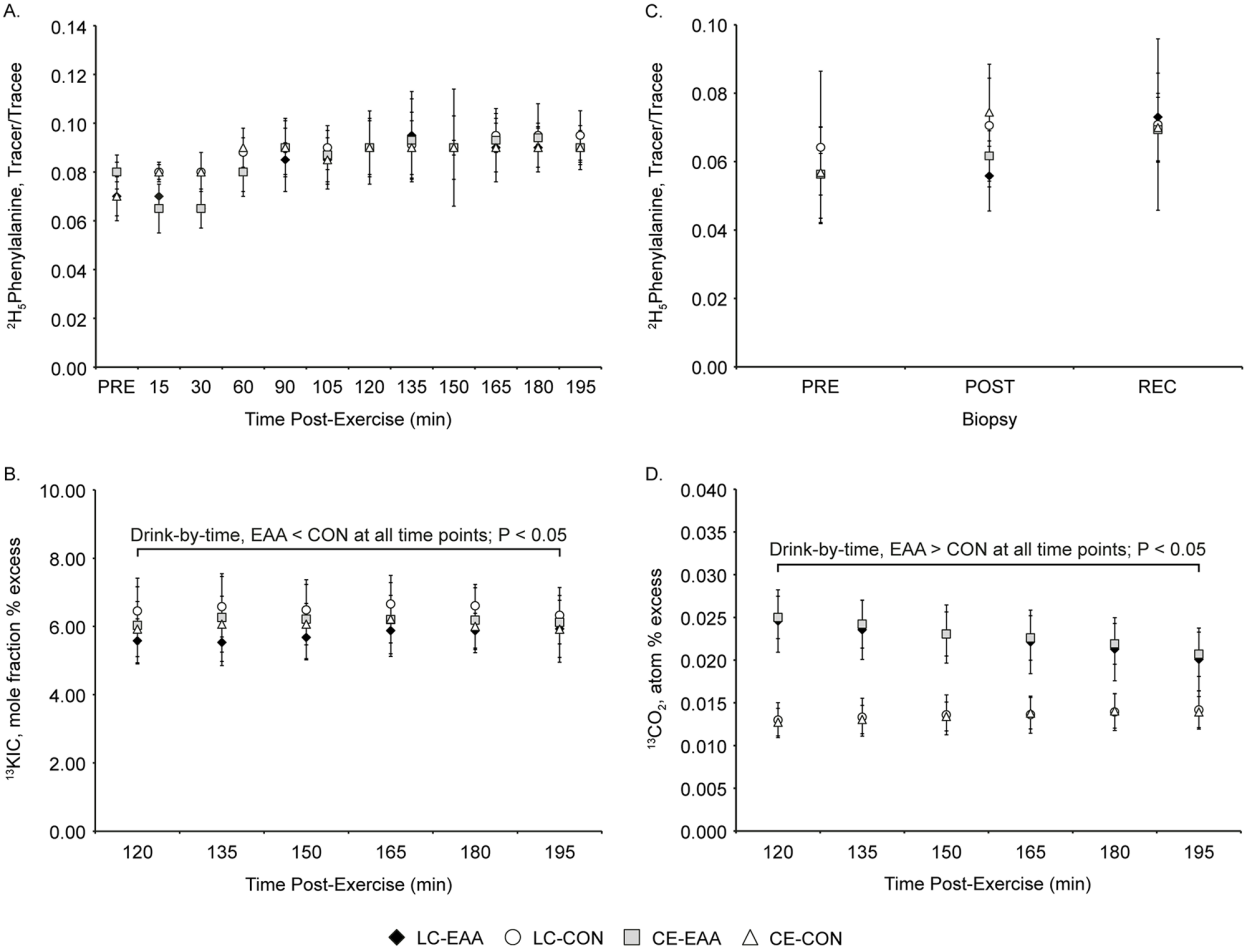
Stable isotope enrichments. Plasma ^2^H_5_-phenylalanine (A), plasma ^13^KIC (B), muscle intracellular ^2^H_5_-phenylalanine (C), and breath ^13^CO_2_ (D) isotopic enrichments during recovery from a 90 min, metabolically matched load carriage (LC) or conventional endurance (CE) exercise bout, with and without (control, CON) essential amino acid (EAA) supplementation. Data are mean ± SD, n = 10 per group. Drink-by-time interactions were observed, EAA different than CON, *P* < 0.05.

Volunteers consumed equal volumes (500 mL total, 125 mL per serving) of either the EAA or flavor-matched, non-nutritive CON drinks in 30 min intervals, beginning at the start of the exercise session and ending after completing the 90 min bout. The EAA formulation and feeding pattern (10 g EAA: 0.7 g histidine, 0.7 g isoleucine, 3.6 g leucine, 1.2 g lysine, 0.3 g methionine, 1.4 g phenylalanine, 1.0 g threonine, and 1 g valine) was based on our previous work demonstrating a MPS and whole-body protein turnover advantage of consuming small doses of leucine-enriched EAA during CE [1]. The EAA drink also provided 46 g of carbohydrate, which we recognize may contribute to our turnover outcomes. However, our intent was to test a palatable, eat-on-the move, combat ration recovery beverage item that provides not only EAA to optimize MPS and whole-body protein turnover, but also energy in the form of carbohydrate (223 kcal) to sustain activity during military operations. Furthermore, recent data suggests that adding carbohydrate to a 10g EAA solution does not enhance MPS above and beyond consuming the 10 g dose of EAA alone [18]. The phenylalanine and leucine content of the EAA drink were not enriched with small amounts of L-[^2^H_5_]-phenylalanine and L-[1-^13^C]-leucine, an approach commonly used when EAA (or protein) is provided as a bolus to limit the potential dilution of the tracer pool [19]. Consuming the EAA drink in four small doses (i.e., 350 mg of phenylalanine and 900 mg of leucine per serving), over 90 min period, likely minimized any isotopic dilution that may have occurred if the EAA drink was consumed as a bolus (Fig 2A–2D).

The CON drink (22 kcal, 5 g carbohydrate) was similar in taste and appearance, but was essentially void of any nutrition (22 kcal, 5 g carbohydrate) to ensure appropriate comparisons between the exercise modes themselves. Study staff and volunteers were blinded and supplements were prepared and coded by an independent third party (Combat Feeding Directorate, US Army Natick Soldier Systems Center, Natick, MA) to eliminate bias. The EAA premix was purchased commercially (Fortitech, Inc., Schenectady, NY) and nutrient content was confirmed before use (Covance Laboratories, Inc., Madison, WI).

Additional muscle biopsies were obtained from the same incision 15 min post-exercise (POST) and 195 min into recovery (REC). Visible blood and connective tissue were removed from muscle specimens and samples were then frozen in liquid nitrogen. Blood and breath samples were collected throughout recovery to measure isotopic enrichments, whole-body protein turnover, leucine, and insulin (Fig 1).

### Determination of muscle protein synthesis and whole-body protein turnover

Myofibrillar and sarcoplasmic enriched muscle protein subfractions were isolated using methods previously described to examine LC-induced modulations in contractile and metabolic protein synthesis rates as compared to CE [20]. In brief, muscle samples (~50 mg) were homogenized in chilled buffer (10 μL∙mg^-1^) containing 25mM Tris-HCL (pH 7.2), 0.5% Triton-X-100 (Sigma-Aldrich, St. Louis, MO), 1 PhosStop^™^ tablet (Roche, Indianapolis, IN) and 1 complete mini protease inhibitor tab (Roche, Indianapolis, IN) per 10 mL of chilled buffer. Following centrifugation (2200xg for 10 min at 4°C), 300 μL of the supernatant was stored for sarcoplasmic enriched fraction isolation by the addition of 1 mL perchloric acid (the remaining supernatant was stored). The myofibrillar and collagen pellet was washed (500 μL of distilled water) and centrifuged (700xg for 10 min at 4°C). Myofibrillar proteins were solubilized by adding 1 mL of 0.3 M NaOH and heating at 50°C for 30 min, with vortex mixing every 10 minutes. The supernatant containing the myofibrillar enriched fraction was stored and the collagen pellet was discarded. Myofibrillar proteins were precipitated by adding 1 mL of 1M perchloric acid, followed by centrifugation for 10 min at 700xg 4°C. After the sarcoplasmic and myofibrillar enriched fractions were washed and lyophilized, the amino acids were hydrolyzed by adding 2 mL of 6 M HCL and heating overnight at 110°C. Mixed-muscle protein, muscle intracellular free (and plasma) enriched amino acids were also determined using well-documented methods [12]. All enriched components were purified using cation exchange columns (Dowex 50WX8-200 resin, Sigma-Aldrich, St. Louis, MO) and isotopic enrichments were determined using the *t*-BDMS derivative of phenylalanine and gas chromatography (GC)- mass spectrometry (MS; Hewlett Packard 5890 Series II, Palo Alto, CA) analysis of the appropriate mass/charge ratios [20]. Mixed-muscle, myofibrillar, and sarcoplasmic MPS were determined using the precursor-product method:

MPS = Ep2-Ep1/[Eprecursor × t] ×100

for exercise MPS, Ep_2_ and Ep_1_ represent the muscle protein bound enrichments measured in the POST and PRE muscle samples, respectively. For recovery measures of MPS, Ep_2_ and Ep_1_ represent the muscle protein bound enrichments measured in the REC and POST muscle samples, respectively. E_precursor_ is the average muscle intracellular L-[^2^H_5_]-phenylalanine enrichment for the exercise (PRE and POST) and recovery (POST and REC) muscle samples, and *t* indicates the tracer incorporation time [12,21].

Plasma ^13^C-α-KIC and breath ^13^CO_2_ enrichments were determined by GC-MS and isotope ratio-MS, respectively (Metabolic Solutions, Nashua, NH). Isotopic enrichment data from six time points (120, 135, 150, 165, 180, and 195 min post-exercise) were corrected for baseline enrichments and used to confirm isotopic steady state. Whole-body protein turnover was calculated in the later stages of recovery to be consistent with our previous work and to ensure isotopic steady state was achieved [1]. Steady state conditions were assumed when the CV between time points were ≤ 10% [22]. These data were used to calculate whole-body protein turnover (Flux) [23]:

Flux=i(Ei/Ep)

where *i* represents the infusion rate of L-[1-^13^C] leucine, *E* is the isotope enrichment, *E*i is the ^13^C enrichment of the L-[1-^13^C] leucine infusate, and *E*p is the plasma ^13^C-α-KIC enrichment. Whole-body protein breakdown was calculated as the difference between Flux (minus the tracer infusion rate) and leucine intake during the post-prandial period (EAA leucine intake, 65 ± 9 μmol∙kg^-1^∙hr^-1^). The rate of leucine oxidation was calculated from the ^13^CO_2_ excretion rate and the plasma ^13^C-α-KIC enrichment:

Oxidation =F13CO2/(R ×Ep)×100

*F*_13CO2_ represents the ^13^CO_2_ excretion rate, R is the fractional bicarbonate retention factor (i.e., the fraction of ^13^CO_2_ released from L-[1-^13^C] leucine oxidation and present in expired breath), and *E*p is plasma ^13^C-α-KIC enrichment. The values of 0.70 and 0.83, respectively, were used for *R* in the postabsorptive and postprandial states [24]. Whole-body protein synthesis was calculated as flux minus oxidation (i.e., non-oxidative leucine disposal) and net protein balance was determined as the difference between total leucine intake (including the tracers) and oxidation.

### Amino acids, insulin, surrogate markers of muscle damage, and soreness analysis

Plasma amino acids (i.e., EAA, branched-chain amino acids [BCAA], and leucine) concentrations were determined using high-performance liquid chromatography and o-phthaldialdehyde post column derivatization (Agilent 1100 Series HPLC, Agilent Technologies, Foster City, CA). Plasma insulin concentrations were determined using an advanced automated immunoassay instrument (Immulite^®^ 2000: Siemens Healthcare Diagnostic, Deerfield, IL). EAA, BCAA, leucine, and insulin concentrations were determined at baseline, 15, 30, 60, 90, 150, and 195 min post-exercise. Subjective ratings of muscle soreness (deltoids, quadriceps, gluteus, and gastrocnemius/soleus) were collected from participants immediately (within 15 min post-exercise) and 195 min post-exercise using a validated visual analogue scale; results were reported as a percentage, with higher scores indicating greater soreness [25]. Circulating surrogates of muscle damage were also assessed, but only at baseline and 195 min post-exercise, including creatine kinase (CK), lactate dehydrogenase (LDH; Beckman Coulter DXC 600 Pro, Beckman Coulter, Brea CA), and myoglobin (Siemens Immulite 2000, Siemens Medical Solutions USA Inc., Malvern, PA).

### Statistical analyses

Baseline volunteer characteristics are described using common descriptive statistics (Table 3). A one-way ANOVA was used to confirm homogeneity between groups. Univariate ANOVA was used to determine main and interactive effects of exercise mode (LC vs. CE) and drink (EAA vs. CON) during exercise and recovery. Whole-body protein flux, synthesis, breakdown, oxidation, and net balance were assessed using a univariate ANOVA to determine main effects of exercise mode, dietary treatment, and their interactions. A mixed-model ANOVA was used to determine main effects of exercise mode, dietary treatment, time, and their interactions for amino acids and insulin. Bonferroni adjustments were conducted to adjust for multiple *post hoc* comparisons if significant interactions were observed. The alpha level for significance was set at *P* < 0.05. All data were analyzed using SPSS (Version 21.0, 2010, SPSS Inc, Chicago, IL) and expressed as means ± SD.

**Table 3:** Volunteer characteristics.

	CE		LC	
	CON	EAA	CON	EAA
Age (y)	22 ± 4	22 ± 2	24 ± 5	22 ± 3
Height (cm)	175 ± 8	177 ± 7	177 ± 8	178 ± 5
Body mass (kg)	78 ± 11	84 ± 10	77 ± 10	81 ± 10
Fat mass (kg)	17 ± 5	20 ± 3	17 ± 5	17 ± 5
Fat-free mass (kg)	62 ± 9	65 ± 9	61 ± 9	64 ± 6
Body fat (%)	22 ± 5	24 ± 4	22 ± 6	22 ± 4
VO_2peak_ (mL∙kg^-1^∙min^-1^)	50 ± 4	49 ± 4	51 ± 5	51 ± 4

Data are mean ± SD, n = 10 per group. CE, conventional endurance exercise; LC, load carriage; CON, control; and EAA, essential amino acids.

## Results

**Fig 3:**
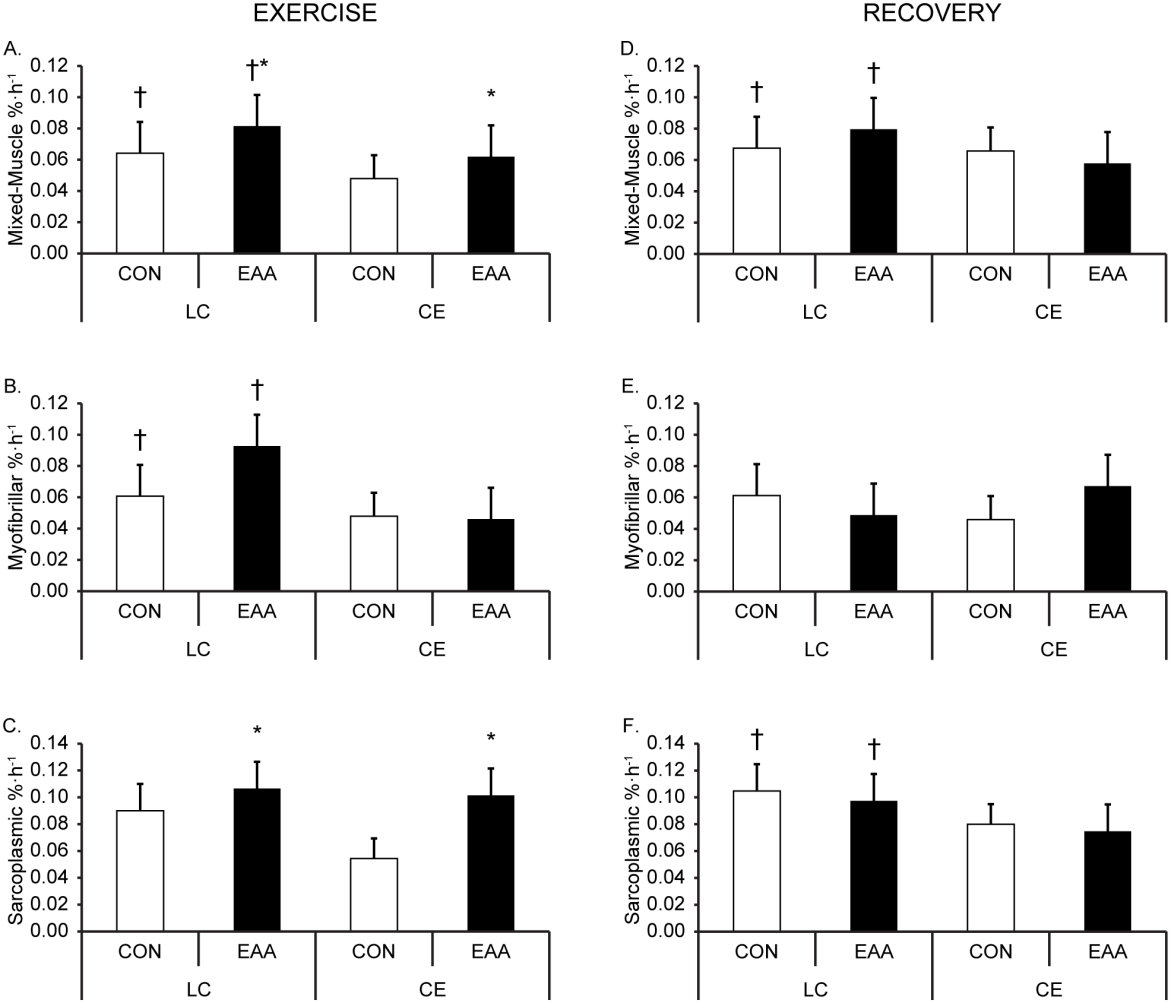
Protein synthesis. Mixed-muscle (A), myofibrillar (B), and sarcoplasmic (C) muscle protein synthesis (MPS) during exercise and mixed-muscle (D), myofibrillar (E), and sarcoplasmic (F) MPS in recovery from a 90 min, metabolically matched load carriage (LC) or conventional endurance (CE) exercise bout, with and without (control, CON) essential amino acid (EAA) supplementation. Data are mean ± SD, n = 10 per group. ^†^Mode main effect; LC different than CE, *P* < 0.05. *Drink main effect; EAA different than CON, *P* < 0.05.

Mixed-muscle and myofibrillar MPS were 31% and 56% higher during exercise for LC compared to CE (mode main effect, *P* < 0.05, Fig 3A and 3B). EAA upregulated mixed-muscle and sarcoplasmic MPS during exercise, regardless of exercise mode (drink main effect, *P* < 0.05, Fig 3A and 3C). There were no interactions between exercise mode (LC and CE) and drink (EAA and CON) on mixed-muscle, myofibrillar, and sarcoplasmic MPS during exercise.

Mixed-muscle MPS was higher in recovery for LC compared to CE (mode main effect, *P* < 0.05, Fig 3D). Myofibrillar MPS was not different between modes in recovery (Fig 3E). However, sarcoplasmic MPS was 30% higher in recovery after LC than CE (mode main effect, *P* < 0.05, Fig 3F). There were no differences between EAA and CON in recovery, nor were there interactions between exercise mode (LC and CE) and drink (EAA and CON) on any MPS measure.

**Fig 4:**
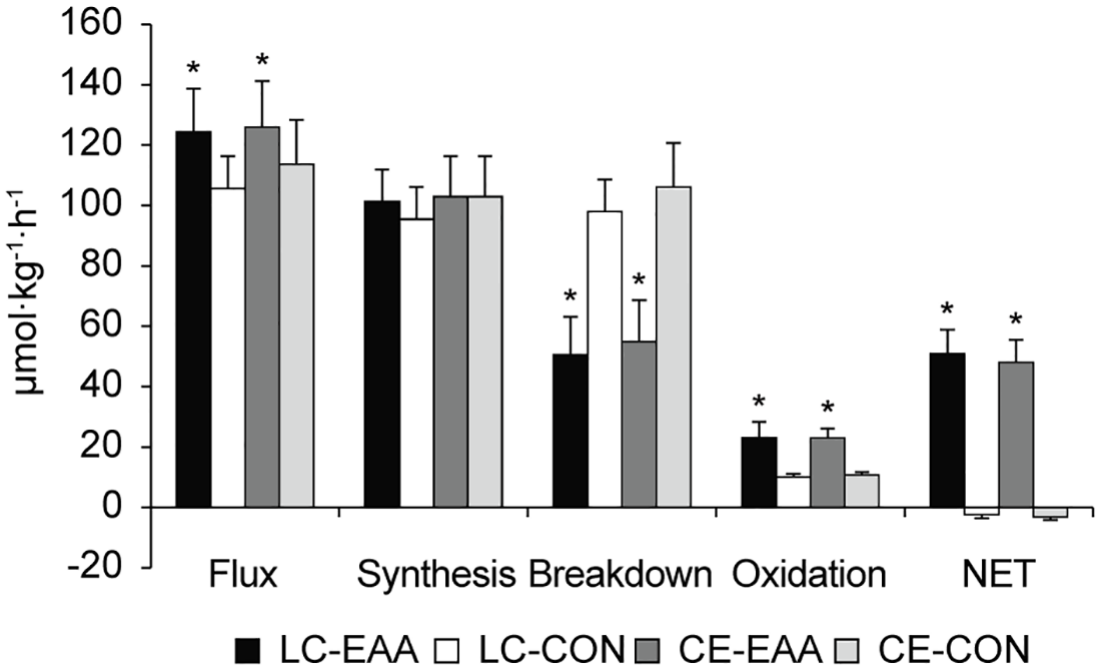
Whole-body protein turnover. Flux, synthesis, breakdown, oxidation, and net protein balance during recovery from a 90 min, metabolically matched load carriage (LC) or conventional endurance (CE) exercise bout, with and without (control, CON) essential amino acid (EAA) supplementation. Data are mean ± SD, n = 10 per group. *Drink main effect; EAA different than CON, *P* < 0.05.

Whole-body protein flux when measured during recovery was 15% higher (drink main effect, *P* < 0.05, Fig 4) when EAA were consumed during exercise. EAA supplementation attenuated whole-body protein breakdown, increased oxidation, and enhanced net protein balance compared to CON (drink main effect, *P* < 0.05). Protein synthesis was not different between groups nor were there differences between LC and CE for any whole-body protein turnover measure.

**Fig 5:**
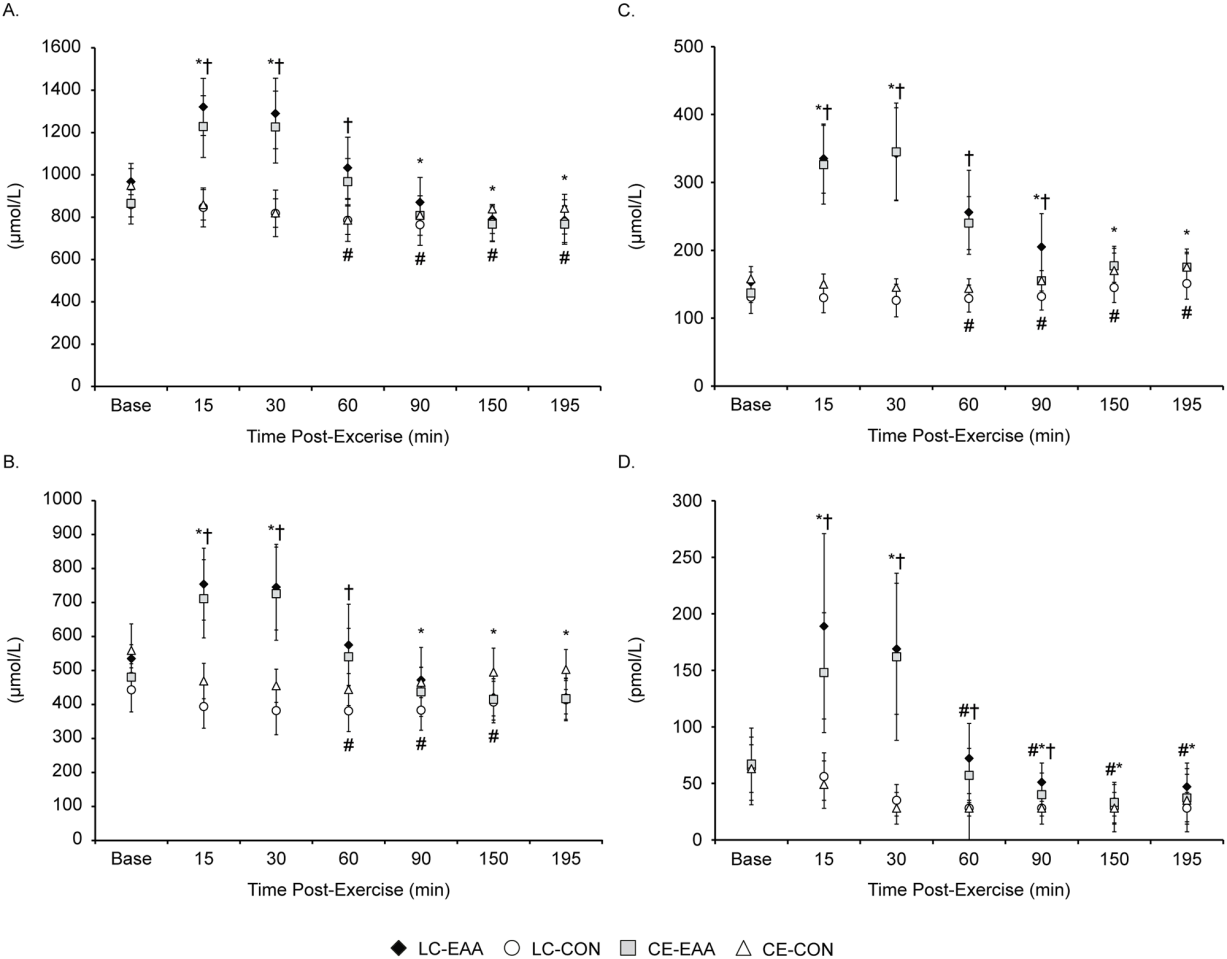
Amino acids and insulin. Effects of load carriage, conventional endurance exercise, and essential amino acid supplementation on plasma essential amino acids (A), branched-chain amino acids (B), leucine (C), and insulin (D) during recovery. Data are mean ± SD, n = 10 per group. CE, conventional endurance exercise; LC, load carriage; CON, control; and EAA, essential amino acids. Drink x time (D x T) interaction for EAA, BCAA (branched-chain amino acids), leucine, and insulin. *Different from baseline for EAA groups, ^#^from baseline for CON groups, and for EAA groups compared to ^†^CON, D x T, *P* < 0.05.

EAA, BCAA, leucine, and insulin concentrations during recovery after LC and CE were higher for EAA than CON (drink x time, *P* < 0.05, Fig 5A–5D). More specifically, concentrations were increased above baseline 15 and 30 min post-exercise before returning (60 min) and dropping below baseline levels. Exercise mode did not augment plasma amino acids and insulin responses to exercise and EAA supplementation. Deltoid soreness immediately and 195 min post-exercise was higher for LC than CE (mode x time, *P* < 0.05, Table 4). Overall, quadriceps soreness was higher immediately post-exercise relative to195 min post-exercise and for CE compared to LC (mode and time main effects, *P* < 0.05). Gluteus soreness was higher for CE than LC immediately post-exercise and increased over time for CE, whereas there was no change over time in gluteus soreness for LC (mode x time, *P* < 0.05). Gastrocnemius/soleus soreness was higher immediately post-exercise relative to 195 min post-exercise but not different between modes (mode main effect *P* < 0.05). CK was similar between modes and increased 195 min post exercise compared to baseline (mode x time, *P* < 0.05). A small, but significant, increase in LDH occurred in response to both LC and CE, with no differences between modes (time main effect, *P* < 0.05). Overall, myoglobin was higher with LC than CE (mode main effect, *P* < 0.05). LDH and myoglobin tended to be higher 195 min post-exercise for LC than CE (mode x time, *P* = 0.51 and 0.55).

**Table 4:** Effects of load carriage, conventional endurance exercise, and essential amino acid supplementation on surrogate markers of muscle damage and muscle soreness during recovery.

	Baseline	15-min PE	195-min PE	Effect
**Muscle Soreness (%)**				
Deltoids				M x T
LC-EAA	n/a	30.6 ± 31.3^†^	3.2 ± 5.6*	
LC-CON	n/a	28.5 ± 26.7^†^	8.9 ± 16.1*	
CE-EAA	n/a	1.9 ± 3.6	0.7 ± 0.8	
CE-CON	n/a	3.8 ± 6.6	1.1 ± 1.9	
Quadriceps				M, T
LC-EAA	n/a	14.5 ± 8.9	9.2 ± 12.9	
LC-CON	n/a	20.1 ± 16.2	7.2 ± 6.0	
CE-EAA	n/a	33.2 ± 28.4	18.4 ± 26.6	
CE-CON	n/a	31.0 ± 23.8	20.7 ± 19.0	
Gluteus				M x T
LC-EAA	n/a	5.2 ± 8.5^†^	2.7 ± 3.8	
LC-CON	n/a	12.2 ± 21.9^†^	3.6 ± 4.3	
CE-EAA	n/a	35.4 ± 25.8	7.2 ± 7.3*	
CE-CON	n/a	26.5 ± 20.4	1.8 ± 2.4*	
Gastrocnemius/Soleus				
LC-EAA	n/a	23.9 ± 23.5	9.4 ± 14.3	NS
LC-CON	n/a	9.8 ± 12.3	6.3 ± 7.6	
CE-EAA	n/a	14.2 ± 14.8	6.3 ± 11.2	
CE-CON	n/a	16.0 ± 13.3	5.9 ± 14.9	
**Muscle Damage**				
Creatine Kinase				
LC-EAA	202.1 ± 157.3	n/a	272.0 ± 85.9*	M x T
LC-CON	209.7 ± 197.5	n/a	269.1 ± 162.5*	
CE-EAA	175.0 ± 169.6	n/a	248.1 ± 169.8*	
CE-CON	178.5 ± 122.1	n/a	275.2 ± 195.6*	
Lactate Dehydrogenase				
LC-EAA	134.8 ± 43.3	n/a	140.3 ± 22.6	T
LC-CON	134.3 ± 27.5	n/a	150.8 ± 22.0	
CE-EAA	126.7 ± 21.5	n/a	136.4 ± 25.4	
CE-CON	121.5 ± 29.5	n/a	137.8 ± 21.2	
Myoglobin				
LC-EAA	29.8 5.4	n/a	68.5 25.4	M
LC-CON	28.9 11.6	n/a	109.3 85.9	
CE-EAA	24.8 8.2	n/a	56.4 29.3	
CE-CON	25.3 6.0	n/a	58.2 21.9	

Data are mean ± SD, n = 10 per group. CE, conventional endurance exercise; LC, load carriage; CON, control; and EAA, essential amino acids; PE, post-exercise. Mode x time (M x T) interaction for deltoids, gluteus, and creatine kinase. Mode (M) and time (T) main effects for quadriceps, lactate dehydrogenase, and myoglobin. M and T effects indicate overall mean difference between modes and time, *P* < 0.05.

*Different from baseline (or 15-min PE) within mode and

^†^corresponding time point for CE, M x T, *P* < 0.05.

## Discussion

The major finding from this randomized, double-blind, placebo controlled study is that LC produces an anabolic stimulus that upregulates mixed-muscle and myofibrillar MPS of the vastus lateralis during exercise and sarcoplasmic MPS during recovery to a greater extent than an absolute VO_2_-matched bout of CE. We also found that consuming EAA produced elevations in MPS during exercise that were independent of exercise mode and specific to mixed-muscle and the sarcoplasmic protein subfraction. Consuming EAA during LC and CE enhanced whole-body protein balance in recovery to the same extent, independent of exercise mode. The findings from this study suggest that when the oxygen and energy cost of LC and CE are the same, whole-body protein turnover in recovery may be the same, but LC appears to produce a local muscle protein synthetic stimulus that exceeds CE.

LC produced a greater muscle protein synthetic response than CE during exercise. The greater mixed-muscle and myofibrillar protein synthetic responses to LC are consistent with our hypothesis and suggest that the resistive-type contractile forces generated during LC elicit an anabolic stimulus that is higher than CE. It is plausible that LC elicited a general increase in protein turnover to support muscle remodeling, accounting for the observed differences between modes during exercise for mixed-muscle and myofibrillar MPS. Although measurements of muscle protein breakdown or molecular biomarkers of muscle proteolysis would have strengthened this assertion, others have also hypothesized that muscle remodeling elicits a general increase in protein turnover that is reflected in myofibrillar MPS [26]. We did not anticipate sarcoplasmic MPS during recovery being higher for LC than CE, largely because the energy cost and aerobic workloads were matched. These findings may be due to the mitochondrial content of the non-myofibrillar, sarcoplasmic subfraction, given the methods we employed to isolate the sarcoplasmic subfraction do not differentiate mitochondrial proteins from the non-myofibrillar enriched protein fraction [20]. That said, it is also difficult to compare our findings with the current literature, particularly because no studies have used LC as an exercise model in protein turnover studies. We suspect that matching the energy cost and aerobic stimulus isolated the mechanical loading differences between LC and CE, producing an environment that, to an extent, resembles mixed-muscle, myofibrillar, and sarcoplasmic MPS responses to low-load, high volume resistance exercise [20,27] and sustained mechanical loading [28]. Perhaps the most appropriate comparison to LC is the protein synthetic response to resistance exercise and concurrent CE [13,14]. For example, respective changes in myofibrillar and mitochondrial MPS after concurrent submaximal leg extensions and cycle ergometry are equivalent to the anabolic response to either mode of exercise performed alone [13]. Our findings suggest that the combined mechanical and aerobic stimulus of LC upregulates the synthesis of both contractile and oxidative proteins in a similar pattern to performing low-load, high volume resistance exercise that maximizes muscle time under tension or resistance exercise and CE in tandem.

EAA supplementation increased mixed-muscle and sarcoplasmic MPS during LC and CE; findings consistent with the established anabolic properties of EAA on skeletal muscle [29]. However, we hypothesized that the anabolic response to EAA and LC would be additive, particularly in the myofibrillar protein subfraction. Myofibrillar MPS during exercise was numerically the highest when EAA were consumed during LC, but there was no statistical interaction between exercise mode and dietary treatment. Sarcoplasmic MPS responses to EAA during exercise were also not affected by mode.

The effects of EAA on MPS are entirely plausible in the context of our intermittent feeding and 3 h recovery protocol. The lack of a more apparent synergistic anabolic effect of LC and EAA on myofibrillar and sarcoplasmic MPS is also similar to an earlier study showing no additive effect of protein supplementation on myofibrillar and sarcoplasmic MPS measured 3 h into recovery from resistance exercise [30]. The inability for EAA supplementation to produce measureable differences in MPS within or between exercise modes is likely attributed to similar declines in post-exercise extracellular leucine availability across groups. It is also possible that MPS responses to EAA provisions during the exercise bout were underestimated, as the precursor pool may have been diluted by the feeding protocol, although our post-exercise precursor enrichments would argue against this point. Regardless, we suspect that if we had used a bolus rather than intermittent EAA supplementation scheme our findings would have been different [31,32].

There are experimental design limitations that need to be considered when interpreting the MPS data. Muscle biopsies were limited to once before exercise and twice after exercise. Performing additional muscle biopsies after exercise may have added resolution to better appreciate interactions between exercise mode and dietary treatment. In addition, we may have underestimated the magnitude by which EAA supplementation stimulated MPS by measuring synthesis over a 3 h period, because MPS generally peaks within the first 60–90 min after consuming EAA and because the exercise intensity was, at the very most, moderate [31]. To be consistent with our previous studies [1,16,33,34], additional muscle biopsies were not included. We also recognize that consuming EAA as a bolus, and not intermittently, may have stimulated a greater increase in MPS than we observed [35], although recent findings suggest that there are no differences in MPS between feeding patterns [36]. Regardless, our intent was not to compare bolus to intermittent feeding, but to evaluate the anabolic stimulus of EAA when consumed as an eat-on-the-move food product during an exercise mode that may contribute to protein loss [5].

Increasing amino acid availability during endurance-type exercise enhances protein balance in recovery by attenuating protein breakdown [1]. The protein-sparing benefit conferred by consuming protein during exercise has been shown by others [22,37]. Dietary amino acids are preferentially oxidized during exercise, thereby limiting oxidation of endogenous protein and promoting positive protein balance in recovery [22]. Although we cannot rule out the potential contribution of the carbohydrate content of the EAA supplement, our study, in part, confirms the whole-body protein-sparing properties of EAA (and/or protein) supplementation during exercise, and despite divergent MPS responses to LC and CE, the mechanical strain of LC does not modulate whole-body protein turnover, at least when measured in recovery from exercise. These findings make sense considering that the contribution of skeletal muscle protein turnover to whole-body protein turnover is relatively small and may be lost when whole-body protein turnover was measured over the last hour of a 3 h recovery period [38]. Nevertheless, our findings suggest that, at the whole-body protein level, LC is no different than CE, with and without EAA, when the energy cost and aerobic workloads are the same.

## Conclusions

In conclusion, this randomized, double-blind, placebo controlled study showed that LC elicits a more robust protein synthetic signal than CE, suggesting CE may be an inappropriate exercise model to evaluate the skeletal muscle protein turnover responses to hiking and other similar activities, particularly military training or combat operations that involve carrying heavy loads [5]. Consuming EAA intermittently during exercise upregulated MPS during exercise and enhanced whole-body protein balance in recovery. Findings from this study provide the basis to examine the magnitude by which protein turnover responses to EAA (and/or protein) spares whole-body protein and skeletal muscle mass during real-world sporting events, occupational tasks, or military and exercise training scenarios that include repeat bouts of weighted exercise.
